# In Vitro Effect of *Taraxacum officinale* Leaf Aqueous Extract on the Interaction between ACE2 Cell Surface Receptor and SARS-CoV-2 Spike Protein D614 and Four Mutants

**DOI:** 10.3390/ph14101055

**Published:** 2021-10-17

**Authors:** Hoai Thi Thu Tran, Michael Gigl, Nguyen Phan Khoi Le, Corinna Dawid, Evelyn Lamy

**Affiliations:** 1 Molecular Preventive Medicine, University Medical Center and Faculty of Medicine, University of Freiburg, 79108 Freiburg, Germany; hoai.tran@uniklinik-freiburg.de (H.T.T.T.); phan.khoi.nguyen.le@uniklinik-freiburg.de (N.P.K.L.); 2 Food Chemistry and Molecular Sensory Science, Technical University of Munich, 85354 Freising, Germany; michael.gigl@tum.de (M.G.); corinna.dawid@tum.de (C.D.)

**Keywords:** ACE2 binding inhibitor, COVID-19, dandelion, SARS-CoV-2 prevention, S1 spike mutation

## Abstract

To date, there have been rapidly spreading new SARS-CoV-2 “variants of concern”. They all contain multiple mutations in the ACE2 receptor recognition site of the spike protein, compared to the original Wuhan sequence, which is of great concern, because of their potential for immune escape. Here we report on the efficacy of common dandelion (*Taraxacum officinale*) to block protein–protein interaction of SARS-COV-2 spike to the human ACE2 receptor. This could be shown for the wild type and mutant forms (D614G, N501Y, and a mix of K417N, E484K, and N501Y) in human HEK293-hACE2 kidney and A549-hACE2-TMPRSS2 lung cells. High-molecular-weight compounds in the water-based extract account for this effect. Infection of the lung cells using SARS-CoV-2 spike D614 and spike Delta (B.1.617.2) variant pseudotyped lentivirus particles was efficiently prevented by the extract and so was virus-triggered pro-inflammatory interleukin 6 secretion. Modern herbal monographs consider the usage of this medicinal plant as safe. Thus, the in vitro results reported here should encourage further research on the clinical relevance and applicability of the extract as prevention strategy for SARS-CoV-2 infection in terms of a non-invasive, oral post-exposure prophylaxis.

## 1. Introduction

In late 2019, the disease known as Corona Virus Disease 2019 or COVID-19 was first reported [1]. It is induced by the severe acute respiratory syndrome coronavirus 2 (SARS-CoV-2). Dry cough, fever, fatigue, headache, myalgias, and diarrhea are common symptoms of the disease. In severe cases people may become critically ill with acute respiratory distress syndrome [2]. The SARS-CoV-2 virus surface is covered by a large number of glycosylated S proteins, which consist of two subunits, S1 and S2. The S1 subunit recognizes and attaches to the membrane-anchored carboxypeptidase angiotensin-converting enzyme 2 (ACE2) receptor on the host cell surface through its receptor binding domain (RBD). The S2 subunit plays a key role in mediating virus–cell fusion and in concert with the host transmembrane protease serine subtype 2 (TMPRSS2), promotes cellular entry [3]. This interaction between the virus and host cell at entry site is crucial for disease onset and progression.

To date, there have been rapidly spreading new variants of SARS-CoV-2, Alpha (variant B.1.1.7), Beta (variant B.1.351), and Gamma (variant P.1). Delta (variant B.1.617.2) recently emerged and spread explosively, replacing Alpha around the world. Most of these variants share the mutation N501Y in the spike protein [4] and SARS-CoV-2 variants with spike protein D614G mutations now predominate globally. The Beta variant contains, besides D614G, other spike mutations, including three mutations (K417N, E484K, and N501Y) in the RBD [5]. Preliminary data suggest a possible association between the observed increased fatality rate and the mutation D614G and it is hypothesized that a conformational change in the spike protein results in increased infectivity [6]. Free energy perturbation calculations for interactions of the N501Y and K417N mutations with both the ACE2 receptor and an antibody derived from COVID-19 patients raise important questions about the possible human immune response and the success of already-available vaccines [7]. Further, increased resistance of the variants Beta and Alpha to antibody neutralization has been reported; for the Beta variant this was largely due to the E484K mutation in the spike protein [8]. The Delta variant has been associated with more severe disease, increased transmission, and breakthrough infections in vaccinated individuals [9,10,11,12]. Liu et al. [13] found that the variants’ spike P681R mutation augments spike processing, which leads to enhanced SARS-CoV-2 fitness over the Alpha variant.

Interference with the interaction site between the spike S1 subunit and ACE2 has the potential to be a major target for therapy or prevention [14]. Compounds of natural origin may offer here some protection against viral cell entry while having no or few side effects. Here we report on the inhibitory potential of dandelion on the binding of the spike S1 protein RBD to the hACE2 cell surface receptor and compare the effect of the original D614 spike protein to its D614G, N501Y, and mix (K417N, E484K, and N501Y) mutations.

*Taraxacum officinale* (L.) Weber ex F.H.Wigg. (common dandelion) belongs to the plant family Asteraceae, subfamily Cichorioideae with many varieties and microspecies. It is a perennial herb, widely distributed in the warmer temperate zones of the Northern Hemisphere inhabiting fields, roadsides, and rural sites. *T. officinale* is consumed as food, but also used in European phytotherapy for diseases of the liver, gallbladder, and digestive tract or for rheumatic diseases. Modern herbal monographs consider the plant usage as safe and have evaluated the empirical use of *T. officinale* for gallstones or biliary diseases with a positive outcome. Therapeutic indications for the use of *T. officinale* are listed in the German Commission E, the European Scientific Cooperative for Phytotherapy (ESCOP) monographs [15,16] as well as by the British Herbal Medicine Association [17]. The plant contains a wide array of phytochemicals including terpenes (sesquiterpene lactones such as taraxinic acid and triterpenes), phenolic compounds (phenolic acids, flavonoids, and coumarins), and also polysaccharides [18]. The predominant phenolic compound was found to be chicoric acid (dicaffeoyltartaric acid). The other constituents are mono- and dicaffeoylquinic acids, tartaric acid derivatives, flavone, and flavonol glycosides. The roots, in addition to these compound classes, contain high amounts of inulin [19]. Dosage forms include aqueous decoction and infusion, expressed juice of fresh plants, and hydroalcoholic tincture as well as coated tablets from dried extracts applied as monopreparations [20] but also integral components of pharmaceutical remedies. The aim of this study was to investigate whether *T. officinale* aqueous leaf extracts and its high molecular weight components block the interaction of ACE2 receptor and SARS-CoV-2 spike protein.

## 2. Results

### 2.1. Chemical Analysis of T. officinale Leaf Extract

**Figure 1:**
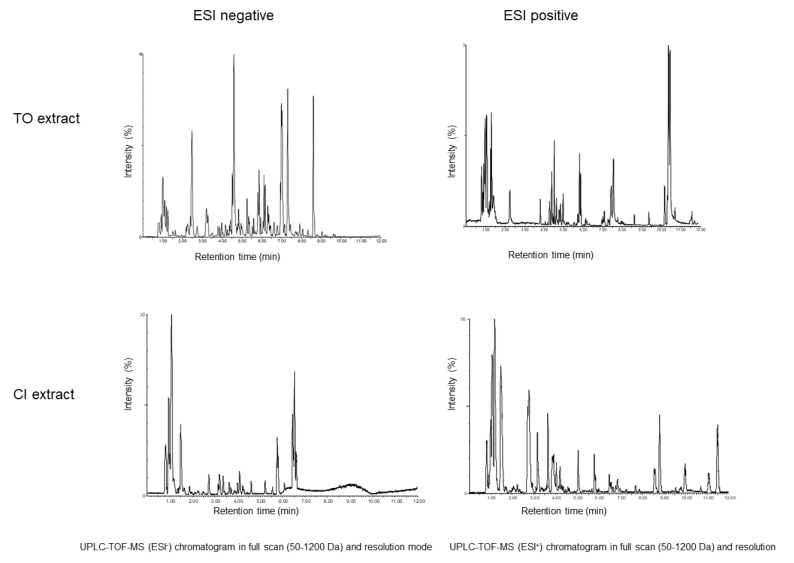
Metabolic analysis of *T. officinale* (TO) and *C. intybus* (CI) leaf extract using UPLC-TOF-MS. Measurements were done in high resolution mode with negative electrospray ionization (ESI−) and positive electrospray ionization (ESI+).

Metabolomics-based fingerprinting of *T. officinale* and *Cichorium intybus L.,* leaf extract was performed by untargeted UPLC-TOF-MS analysis (Figure 1) and putative metabolite identification was done by database search of accurate mass and MS^e^ fragmentation patterns. FoodDB, the Plant Metabolic Network, PlantCyc, and Nature Chemistry were used for tentative annotation. The most abundant compounds of these extracts in both negative ion mode (ESI−) and positive ion mode (ESI+) were identified (Supplementary Tables S1–S4). These are mainly in line with previous reports [18,19,21].

### 2.2. T. officinale Inhibits Spike RBD–ACE2 Binding

**Figure 2:**
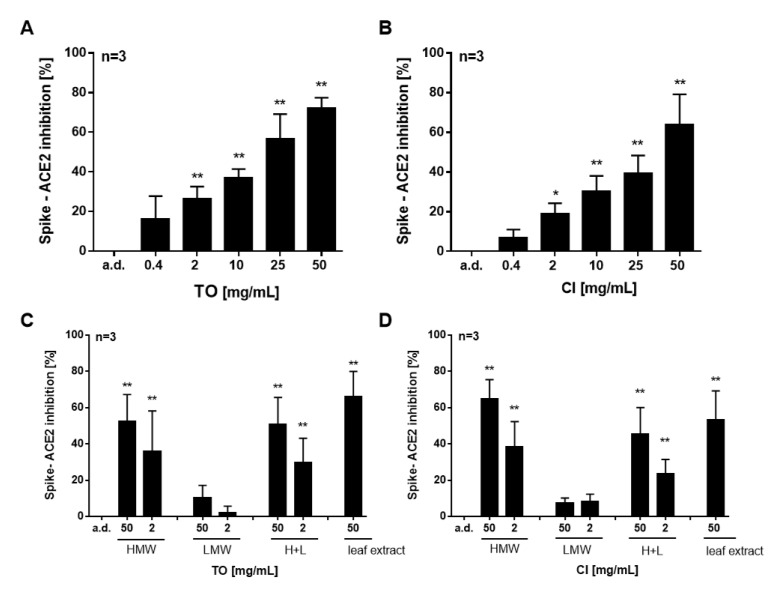
Effect of *T. officinale* and *C. intybus* extracts on SARS-CoV-2-Spike–ACE 2 inhibition. (**A**,**B**) Concentration-dependent effect of *T. officinale* (TO) and *C. intybus* (CI) extract. (**C**,**D**). Effect of fractions from TO and CI leaf extract. The extracts were freeze-dried and a molecular weight fractionation was subsequently carried out. The cut-off was set to 5 kDa (HMW > 5 kDa, LMW < 5kDa). H+L: HMW and LMW fractions; 50 mg of dried leaves per mL water was used as reference. HMW and LMW fraction quantities equivalent to dried leaves were used. The binding inhibition was assessed using ELISA technique. Bars are means + SD. Solvent control: distilled water (a.d.); * *p* < 0.05, ** *p* < 0.01. Significance of difference was calculated relative to the solvent control by one-way ANOVA.

We first investigated the inhibition of interaction between SARS-CoV-2 spike protein RBD and ACE2 using extracts from *T. officinale* leaves. In Figure 2A, the concentration-dependent inhibition of Spike S1–ACE2 binding upon treatment with *T. officinale* extract is given (EC50 = 14.9 mg/mL). Extracts from *C. intybus,* which is another plant from the family Asteraceae, also showed a concentration-dependent binding inhibition, but with less potency (EC50 = 31.4 mg/mL) (Figure 2B). We then prepared two fractions of the extracts, separating them into a high molecular (>5kDa) and low molecular weight (<5kDa) fraction. As can be seen from Figure 2C–D, the bioactive compounds were mostly present in the HMW fraction. Only minor activity was seen in the LMW fraction.

**Figure 3:**
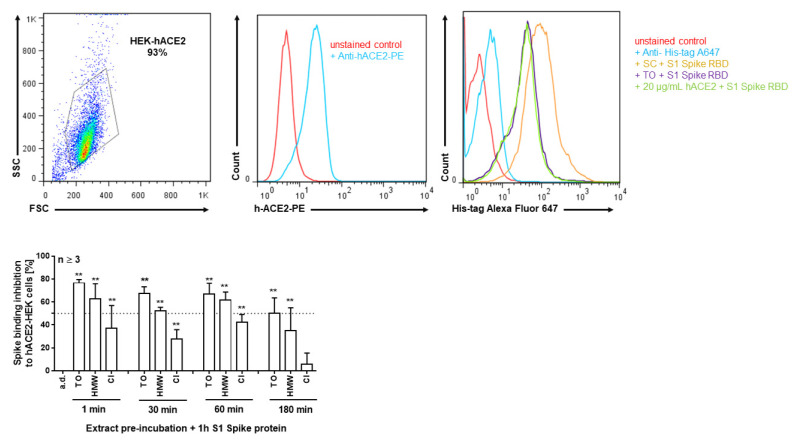
Binding inhibition of S1 spike protein to human HEK293-hACE2 cells by extract pre-incubation. Cells were pre-incubated for the indicated times with 10 mg/mL *T. officinale* (TO), its HMW fraction, equal to 10 mg/mL extract (HMW), and 10 mg/mL *C. intybus* (CI) or solvent control (a.d.) and subsequently treated with His-tagged S1 spike protein for 1 h without a washing step in between at 4 °C. Binding inhibition was assessed using flow cytometry. *N* = 3, bars are means + SD. Upper left: cytogram of gated HEK-hACE2 cells. Middle: overlay of representative fluorescence intensity histograms for ACE2 surface expression. Upper right: overlay of representative fluorescence intensity histograms for spike-binding inhibition by the extracts or a.d.; positive control: 20 µg/mL soluble hACE2. Cells were stained with anti-His-tag Alexa Fluor 647 conjugated monoclonal antibody; ** *p* < 0.01. Significance of difference was calculated relative to the solvent control by one-way ANOVA.

Using hACE2-overexpressing HEK293 cells, the potential of the extracts to block spike binding to cells was further investigated. As can be seen from Figure 3, pre-incubation of cells with *T. officinale* for one minute efficiently blocked cell binding of spike by 76.67% ± 2.9, and its HMW fraction by 62.5 ± 13.4% as compared to the water control. The *C. intybus* extract was less potent; binding inhibition was seen at 37 ± 20% after 1 min.

**Figure 4:**
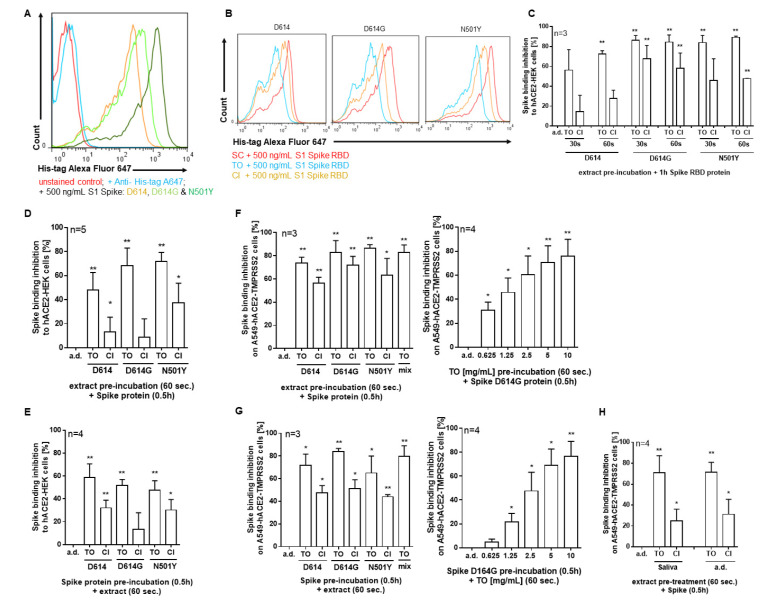
Binding inhibition of spike D614, and its mutants D614G, N501Y or mix (N501Y, K417N and E484K) to human HEK293-hACE2 and A549-hACE2-TMPRSS2 cells by extract pre- or post-incubation. Overlay of fluorescence intensity histogram for (**A**) unstained HEK cells, staining control (anti-His-tag Alexa Fluor 647), and cells incubated with His-tag-labelled spike D614, D614G or N501Y for 1 h at 4 °C. (**B**,**C**) cells pre-incubated with solvent control (a.d.), 10 mg/mL *T. officinale* (TO) or 10 mg/mL *C. intybus* (CI) for 30–60 s, and then treated with His-tag-labelled S1 spike D614, D614G or N501Y protein for 1 h without a washing step in between at 4 °C. (**D**–**G**) Effect of extract incubation on HEK or A549 cells either before or after incubation with His-tag-labelled spike D614, D614G, N501Y or mix (N501Y, K417N and E484K) protein at 37 °C. (**H**) Plant extracts were incubated in saliva from four human donors for 0.5 h at 37 °C. Afterwards, cells were pre-treated with 5 mg/mL extracts for 60 s at 37 °C before incubation with His-tag-labelled spike D614 protein for 0.5 h at 37 °C. Spike-binding inhibition to human cells was assessed using flow cytometric analysis of cells stained with anti-His-tag Alexa Fluor 647 conjugated monoclonal antibody. Bars are means +SD; * *p* < 0.05, ** *p* < 0.01. Significance of difference was calculated relative to the respective solvent control by one-way ANOVA.

Cell treatment with equal amounts of spike D614 and its variants D614G and N501Y confirmed a stronger binding affinity of D614G (about 1.5-fold) and N501Y (about 3- to 4-fold) than D614 spike protein to the ACE2 surface receptor of HEK293 cells (Figure 4A). Pre-treatment with *T. officinale* quickly (within 30 s) blocked spike binding to the ACE2 surface receptor (Figure 4B,C). After 30 s, this was 58.2 ± 28.7% for D614, 88.2 ± 4.6% for D614G, and 88 ± 1.3% for N501Y binding inhibition by *T. officinale* extract. Even though for *C. intybus* extract a binding inhibition of spike could be seen, this was about 30–70% less compared to *T. officinale*, dependent on the spike protein investigated. When binding was studied at 37 °C instead of 4 °C, the results were comparable for *T. officinale*, but even weaker for *C. intybus* extract in this cell line (Figure 4D). We also raised the question of whether the extracts could replace spike binding to the ACE2 surface receptor of human cells. For this, we first incubated the cells with D614, D614G, or N501Y spike protein and subsequently with the extracts. As seen in Figure 4D, *T. officinale* could potently remove spike from the receptor (on average 50%); *C. intybus* was much weaker than (on average 25%). We extended our experiments to human A549-hACE2-TMPRSS2 cells and could confirm the results observed in HEK293-hACE2 cells for *T. officinale* (Figure 4D–G). This cell line has been stably transfected with both the human ACE2 and TMPRSS2 genes and interestingly, here the *C. intybus* extract was more effective as compared to HEK-hACE2 cells. Upon extract pre-treatment, spike-binding inhibition to the cells was between 73.5% ± 5.2 (D614) to 86.3% ± 3.23 (N501Y) for *T. officinale* extract and 56.1% ± 5.28 (D614) to 63.07% ± 14.55 (N501Y) for *C. intybus* extract. Already at 0.6 mg/mL, *T. officinale* significantly blocked binding to D614G spike protein by about 40% (IC50 = 1.73 mg/mL). When cells were pre-incubated with the spike protein before extract treatment, results were comparable for *T. officinale* extract for D614 and D614G but somewhat lower for N501Y (Figure 4C,D). Also, in this setting, a mixture of spike mutants N501Y, K417N, and E484K was tested, and here again, *T. officinale* extract blocked binding by 82.97% ± 6.31(extract pre-incubation) and 79.7% ± 9.15 (extract post-incubation). Extracts, incubated in human saliva for 30 min at 37 °C before cell treatment had comparable effects on spike D614G inhibition (Figure 4H) indicating a good stability of the bioactive compounds in saliva.

### 2.3. T. officinale Does Not Interfere with ACE2 Enzyme Activity

**Figure 5:**
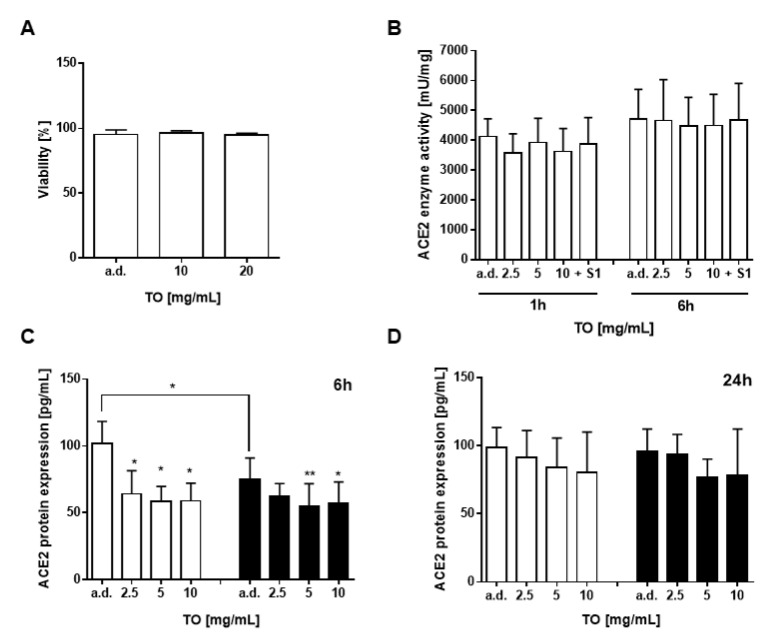
Effect of *T. officinale* extract on ACE2 enzyme activity and protein expression. (**A**) Viability of A549-hACE2-TMPRSS2 cells was determined using trypan blue cell staining after 84 h exposure to the extract. (**B**) Cells were incubated with TO extract or 500 ng/mL S1 protein and analyzed for enzyme activity using a fluorescence kit. (**C**,**D**) Cells were exposed for 6 h or 24 h to extract without (white bars) or with (black bars) 500 ng/mL S1 protein and analyzed for ACE2 protein expression using a human ACE2 ELISA kit; a.d.: solvent control. Bars are means + SD, *N* ≥ 3 independent experiments; * *p* < 0.05, ** *p* < 0.01. Significance of difference was calculated relative to the respective control by one-way ANOVA.

To see whether *T. officinale* extract interferes with the catalytic activity of the ACE2 receptor or affects ACE2 protein expression, we treated A549-hACE2-TMPRSS2 cells with the extract for 1–24 h before cell lysis and detection. No loss in cell viability was seen after extract exposure to the cells for 84 h (Figure 5A). No enzyme activity impairment could be detected after 1 or 24 h (Figure 5B). Spike significantly downregulated ACE2 protein after 6 h (Figure 5C, black bars), and this was also true for the extract, either alone (Figure 5C, white bars) or in combination with spike (black bars). After 24 h, this effect was abolished (Figure 5D).

### 2.4. T. officinale Blocks SARS-CoV-2 Spike D614 and Spike Delta (B.1.617.2) Variant Pseudotyped Lentivirus Transduction

**Figure 6:**
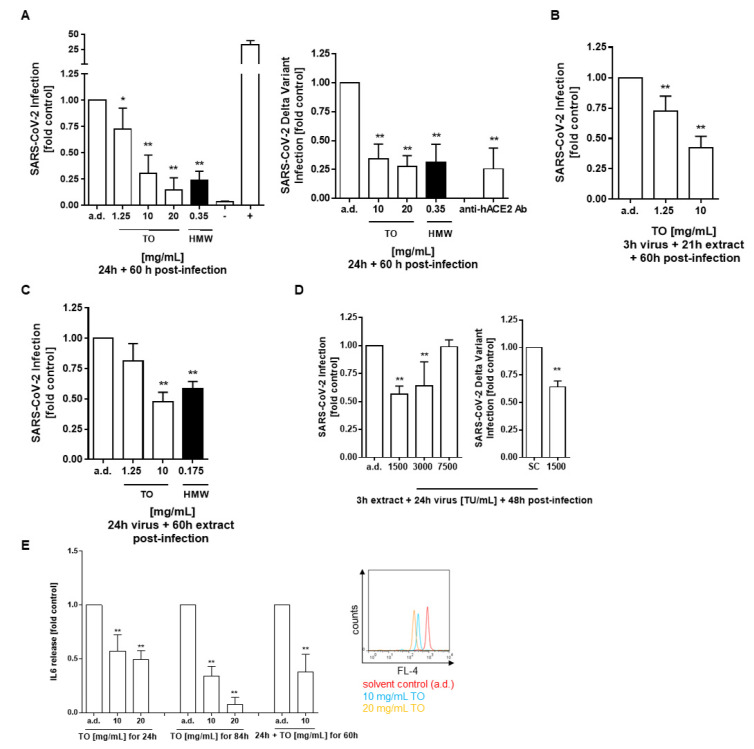
Viral transduction inhibition of A549-hACE2-TMPRSS2 cells by *T. officinale* extract. (**A**) Cells were pre-treated with *T. officinale* (TO) or HMW extract for 0.5 h before infection with 7500 TU/mL SARS-CoV-2 spike D614 or Delta (B.1.617.2) variant for 24 h; (**B**,**C**) Cells were transduced with 7500 TU/mL SARS-CoV-2 for (B) 3 h before addition of TO for another 21 h or (C) 24 h. After transduction, the medium was exchanged with fresh medium containing TO or HMW extract at the indicated concentrations and post-incubated for 60 h. (**D**) Cells were pre-treated with 40 mg/mL TO for 3 h before transduction with the indicated virus titer for 24 h. After that, the medium was exchanged with fresh medium and incubated for another 48 h. Luminescence was then detected within 1 h. 0.35 mg/mL HMW extract equals to 20 mg/mL TO extract. Transduction control: (−) negative control: bald lentiviral pseudovirion; (+) positive control: firefly luciferase lentivirus; inhibitor positive control: 100 µg/mL anti-hACE2 antibody. (**E**) Pro-inflammatory IL-6 cytokine secretion analysis was done either after 24 h virus transduction together with extract (left), after 24 h + 60 h post-infection with extract (middle) or after 60 h post-infection with extract (right) using multiplexing flow cytometric analysis. Solvent control: distilled water (a.d.). N ≥ 3 independent experiments; * *p* < 0.05, ** *p* < 0.01. Significance of difference was calculated relative to the solvent control by one-way ANOVA.

Using a SARS-CoV-2 spike D614 and spike Delta (B.1.617.2) variant pseudotyped lentivirus, we then studied whether the extract could block virus entry via spike inhibition. When pre-treated with the extract, spike D614 virus transduction was diminished by about 85% at 20 mg/mL, which was in the range of inhibition observed by 0.35 mg/mL of the HMW extract (Figure 6A, left). As shown in Figure 6A (right), a substantial reduction in Delta variant pseudovirus infection of lung cells was also observed upon treatment with extracts from *T. officinale* or the HMW extract (72% at 20 mg/mL, and 69% at 0.35 mg/mL, respectively). Anti-hACE2 antibody, which was used here as a reference, blocked virus transduction by 74% at 100 µg/mL.

To investigate which step of the SARS-CoV-2 pseudovirus infection was targeted by the extract, we treated cells at different time points, such as before (pre-treatment), during (co-treatment), or after (post-treatment) viral infection. Under the different treatment conditions, the luminescent signal produced by spike D614 virus transduction was inhibited at 10 mg/mL extract by 70% ± 16.7 (A), 58% ± 9.6 (B), and 53% ± 8.1 (C). Besides, we addressed the effectiveness of infection prevention in cells without post-infection. It can be seen from Figure 6D that, dependent on the initial virus load, infection was diminished by > 43% at 1500 TU/mL, and 35% at 3000 TU/mL, whereas there was a minimal change at 7500 TU/mL. A 36% inhibition was reached by the plant extract in SARS-CoV-2 spike Delta variant transduced cells at 1500 TU/mL (Figure 6D, right). The inhibition of virus transduction by the extract concurred with a significant suppression of the virus triggered inflammatory response, as determined by diminished secretion of the pro-inflammatory cytokine IL-6 in A549-hACE2-TMPRSS2 cells (Figure 6E).

## 3. Discussion

Developing effective prevention and treatment strategies for SARS-CoV-2 infections remains a challenge as new variants emerge that are likely to be more contagious and better able to escape the immune system. Although the first vaccines have now received marketing authorization, challenges in distribution and concerns about durable effectiveness and risk of re-infection remain [22]. Vaccine breakthrough infections have already been reported, especially transmission with the Delta variant [9,11,12]. This suggests a potential waning in the protective effect of the vaccines against Sars-CoV-2 infections [23,24]. Subsequent infections may, though, possibly be milder than the first one. Booster vaccinations to enhance the immune response may be the best strategy for risk reduction. Besides vaccination, blocking the accessibility of the virus to membrane-bound ACE2 as the primary receptor for SARS-CoV-2 target cell entry, represents an alternative strategy to prevent COVID-19. Here, different approaches exist [25], but of course each of these treatment strategies also has its fundamental as well as translational challenges which need to be overcome for clinical utility. Technical hurdles include off-target potential, ACE2-independent effects, stability, or toxicity [25]. Compounds from natural origin could be an important resource here as they have already been well described and many of them have been established as safe. While in silico docking experiments suggested different common natural compounds as ACE2 inhibitors, spike-binding inhibition to ACE2 has not been shown for most of them so far, which might be explained by a lack of complete coverage of ACE2 binding residues by the compounds [26]. However, for glycyrrhizin, nobiletin, and neohesperidin, ACE2 binding falls partially within the RBD contact region and thus, these have been proposed to additionally block spike binding to ACE2 [20]. The same accounts for synthetic ACE2 inhibitors, such as *N*-(2-aminoethyl)-1 aziridine-ethanamine [27]. In contrast, the lipoglycopeptide antibiotic dalbavancin has now been identified as both an ACE2 binder and SARS-CoV-2 spike-ACE2 inhibitor [28]; SARS-CoV-2 infection was effectively inhibited in both mouse and rhesus macaque models by this compound. In addition, with a hydroalcoholic pomegranate peel extract, blocking of spike-ACE2 interaction was shown at 74%, for its main constituents punicalagin at 64%, and ellagic acid at 36%. Using SARS-CoV-2 spike pseudotyped lentivirus infection of human kidney-2 cells, virus entry was then efficiently blocked by the peel extract [29]. In the present study, we could show potent ACE2-spike S1 RBD protein inhibition by *T. officinale* extracts using a cell-free assay and confirmed this finding by demonstrating efficient ACE2 cell surface binding inhibition in two human cell lines. We observed stronger binding of the variants D614G and N501Y to the ACE2 surface receptor of human cells, but all tested variants were sensitive to binding inhibition by *T. officinale*, either used before spike protein exposure or after. To date, several studies indicate that the D614G viral lineage is more infectious than the D614 virus [30]. In addition, the presence of characteristic mutations such as N501Y results in higher infectivity than the parent strain which might be due to a higher binding affinity between the spike protein and ACE2 [31]. So our findings on *T. officinale* extracts could here be important, as with the progression of the pandemic, new virus variants of potential concern will emerge which may also reduce the efficacy of some vaccines or cause increased rates of reinfections. As mentioned above, an issue in the development of products such as prophylaxis for SARS-CoV-2 infection or for slowing the systemic virus spread, is the selectivity towards virus intrusion with low toxicity needed for the host. For current medical indications, no case of overdose by *T. officinale* has been reported [15,17,20]. The recommended dosage is 4–10 g (about 20–30 mg per mL hot water) up to three times per day (Commission E and ESCOP). Based on the information provided by the European Medicines Agency contraindications for the use of *T. officinale* are hypersensitivity to the Asteraceae plant family or their active compounds, liver and biliary diseases, including bile duct obstruction, gallstones and cholangitis, or active peptic ulcer [20]. The plant is a significant source of potassium [32] and thus a warning is given because of the possible risk for hyperkalemia. The use in children under 12 years of age, or during pregnancy and lactation has not been established due to lack of adequate or sufficient data.

While ACE2 enzyme activity was not affected by *T. officinale* extract in the present study, ACE2 protein was transiently downregulated in the ACE2-overexpressing lung cell line. ACE2 plays a key role in SARS-CoV-2 infection, so lowering ACE2 levels could theoretically provide some initial protection [33]. However, this could also affect important cell physiological functions. ACE2 is an important zinc-dependent mono-carboxypeptidase in the renin–angiotensin pathway, critical in impacting the cardiovascular and immune systems. Disruption of the angiotensin II/angiotensin-(1-7) balance by ACE2 enzyme activity inhibition or protein decrease and more circulating angiotensin II in the system, is recognized to promote lung injury in the context of COVID-19 disease [34]. Thus, this observation needs more attention in ongoing studies.

The lung could be assumed to be the primary target of interest but, ACE2 mRNA and protein expression have been found in epithelial cells of all oral tissues, especially in the buccal mucosa, lip, and tongue [35]. These data concur with the observation of very high salivary viral load in SARS-CoV-2-infected patients [36,37]. As an essential part of the upper aerodigestive tract, the oral cavity is thus believed to play a key role in the transmission and pathogenicity of SARS-CoV-2. There is high potential that prevention of viral colonization at the oral and pharyngeal mucosa could be critical for averting further infection to other organs and the onset of COVID-19 [38]. Commercial virucidal mouth-rinses, povidone-iodine at the first place, have thus been suggested to potentially reduce the SARS-CoV-2 virus load in infected persons [39,40], but significant clinical studies do not exist to date [40]. Blocking SARS-CoV-2 virus binding to cells of the oral cavity with *T. officinale* extracts might be tolerable for a consumer, if necessary only for limited periods of time (e.g., product application after contact with infected persons or when being infected). More physiologically relevant in vitro experiments that were carried out by us showed that only short contact times with *T. officinale* extract were necessary for efficient blocking of SARS-CoV-2 spike binding or for removing already-bound spike from the cell surface. Further evidence of relevance was provided by demonstrating effective protection of *T. officinale* against SARS-CoV-2 spike D614 and Delta variant pseudotyped virus infection of human lung cells. The use of pseudotyped viruses does not allow us to assess the contribution of virion properties such as membrane or envelope proteins to cell tropism [35]. However, pseudovirus data are considered a useful tool to document the importance of ACE2 in the spike-protein-mediated steps of cell entry.

## 4. Materials and Methods

### 4.1. Plant Material

The study was carried out using dried leaves from *T. officinale* (vom Achterhof, Uplengen, Germany; batch no. 37259, B370244, and P351756). *C. intybus* was purchased from Naturideen (Germany).

### 4.2. Plant Extraction

Dried plant material was weighted in an amber glass vial (Carl Roth GmbH, Germany) and mixed with HPLC-grade water (a.d.), at room temperature (RT). Extracts were then incubated for 1 h and centrifuged at 16,000 g (3 min, RT). The supernatant was filtered (0.22 µm) prior to use for the experiments.

### 4.3. Ultra-Performance Liquid Chromatography (UPLC)-Time-of-Flight (TOF)-Mass Spectrometry

High resolution mass spectra were recorded on a Waters Synapt G2-S HDMS mass spectrometer (Waters, Manchester, UK) coupled to an Acquity UPLC core system (Waters, Milford, MA, USA) consisting of a binary solvent manager, sample manager, and column oven. For screening of extracts, aliquots (5 µL) of all samples were injected into the UPLC-TOF-MS system using a BEH C18 column (150 mm × 2.1 mm, 1.7 µm, Waters), operated at a flow rate of 0.4 mL/min at 45 °C. Chromatographic separation was done with a gradient of aqueous formic acid (0.1%, A) and acetonitrile (0.1%, B). The gradient started with a mixture of 1% B and was kept isocratic for 1 min, then, increased to 100 % B within 9 min and held at 100 % B for 1 additional min. Measurements were performed in high resolution mode with negative electrospray ionization (ESI−) and positive electrospray ionization (ESI+). The ion source parameters were as follows: capillary voltage +2.5 kV (ESI+) or -2.5 kV (ESI−), sampling cone 50 V, source offset 30 V, source temperature 150 °C, desolvation temperature 450 °C, cone gas 2 L/h, nebulizer gas flow 6.5 bar, and desolvation gas 850 L/h. Data processing was handled with MassLynx 4.1 (Waters) and Progenisis QI (Waters).

### 4.4. Cell Lines and Culture Conditions

Human embryonic kidney 293 (HEK293) cells, stably expressing hACE2, were generously provided by Prof. Dr. Stefan Pöhlmann (Göttingen, Germany). The cells were maintained in Dulbecco’s modified Eagle medium (DMEM), high glucose supplemented with 10% fetal bovine serum (FBS), 100 U/mL penicillin/streptomycin, and 50 µg/mL zeocin (Life Technologies, Darmstadt, Germany). Human A549-hACE2-TMPRSS2 cells, generated from the human lung A549 cell line were purchased from InvivoGen SAS (Toulouse Cedex 4, France) and maintained in DMEM, high glucose supplemented with 10% heat-inactivated FBS, 100 U/mL penicillin/streptomycin, 100 µg/mL normocin, 0.5 µg/mL puromycin, and 300 µg/mL hygromycin. To subculture, all cells were first rinsed with phosphate-buffered saline (PBS) then incubated with 0.25% trypsin-EDTA until detachment. All cells were cultured at 37 °C in a humidified incubator with 5% CO2/95% air atmosphere.

### 4.5. Analysis of SARS-COV2 Spike–ACE2 Interaction Inhibition Using ELISA and Flow Cytometry

A commercially available SARS-CoV-2 Inhibitor Screening Kit (Cat#: 16605302, Fisher Scientific GmbH, Schwerte, Germany) was used for cell free detection of SARS-CoV-2 Spike–ACE2 interaction inhibition. This colorimetric ELISA assay measures the binding between immobilized SARS-CoV-2 spike protein RBD and biotinylated human ACE2 protein. The colorimetric detection is done using streptavidin-HRP followed by TMB incubation. A SARS-CoV-2 inhibitor (hACE2) was used as method verified reference. % inhibition was calculated relative to the solvent control (distilled water, a.d.).

Cell surface expression of ACE2 was determined by using a human ACE2 PE-conjugated antibody (Bio-Techne GmbH, Wiesbaden-Nordenstadt, Germany) and flow cytometric analysis. For analysis of SARS-CoV-2 S1 Spike RBD–ACE2 binding, 2 × 10^5^ cells (5 x 10^6^ cells/mL) were pre-treated with plant extracts for different time points. Then, 500 ng/mL SARS-CoV-2 Spike S1 (Trenzyme GmbH, Konstanz, Germany), spike S1 D614G, N50Y, or a mix of K417N, E484K, and N501Y (Sino Biological Europe GmbH, Eschborn, Germany)–His recombinant protein were added into each sample, and samples were further incubated for 30–60 min. In another setting, cells were pre-treated with 500 ng/mL SARS-CoV-2 Spike–His recombinant protein for 30 min prior to incubation with the plant extract for 30–60 s at 4 °C or 37 °C. The samples were incubated in PBS buffer containing 5% FBS. Cells were then washed one time with PBS buffer containing 1% FBS at 500g, 5 min before staining with His-tag A647 mAb (Bio-Techne GmbH, Wiesbaden-Nordenstadt, Germany) for 30 min at RT. Subsequently, cells were washed twice as described above. The cells were analyzed by using a FACSCalibur (BD Biosciences, Heidelberg, Germany); 10,000 events were acquired. The median fluorescence intensity (MFI) of each sample were determined using FlowJo software (Ashland, OR, USA). % spike-binding inhibition was calculated relative to the solvent control (distilled water, a.d.).

### 4.6. Human ACE2 Enzyme Activity and ProteinQquantification

A549-hACE2-TMPRSS2 (2 × 10^5^) cells were seeded in a 24-well plate in high glucose DMEM medium, containing 10% heat-inactivated FBS, at 37 °C, 5 % CO_2_. Cells were then treated with *T. officinale* extract with/without 500 ng/mL SARS-CoV-2 S1 Spike RBD protein for 1–24 h. Afterwards, cells were washed with PBS and lysed. 25 µg protein were used for quantification of ACE2 protein (ACE2 ELISA kit), 5 µg for ACE2 enzyme activity (ACE2 activity assay kit, Abcam, Cambridge, UK) according to the manufacturer’s instructions.

### 4.7. Infection of A549-hACE2-TMPRSS2 Cells Using SARS-CoV-2 Spike D614 and Delta (B.1.617.2) Variant Pseudotyped Lentivirus

SARS-CoV-2 spike D614 and Delta variant pseudotyped lentivirus particles, produced with SARS-CoV-2 spike and SARS-CoV-2 B.1.617.2 variant spike (Genbank Accession #QHD43416.1) as the envelope glycoproteins instead of the commonly used VSV-G, were purchased from BPS Bioscience, (Catalog#: 79942, and Catalog #78215, respectively, Biomol, Hamburg, Germany). These pseudovirions also contain the firefly luciferase gene driven by a CMV promoter. Thus, the spike-mediated cell entry can be quantified via luciferase reporter activity. The bald lentiviral pseudovirion (BPS Bioscience #79943), where no envelope glycoprotein is expressed, was used as a negative control. The firefly luciferase lentivirus (Puromycin) from BPS Bioscience, (catalogue#: 79692-P) was used as a positive control for transduction. These viruses constitutively express firefly luciferase under a CMV promoter. Anti-hACE2 antibody (Biomol, NSJ-F49433) was used as reference for transduction inhibition assay. Lung cells were seeded at 1 x 10^5^ cells/cm^2^ in a 96-well plate in DMEM containing 10% heat-inactivated FBS, 100 U/mL penicillin/streptomycin, 100 µg/mL normocin, 0.5 µg/mL puromycin, and 300 µg/mL hygromycin and left overnight. The medium was replaced by DMEM containing 10% heat-inactivated FBS and cells were either pre-treated with *T. officinale* extract at different time points before adding the lentivirus particles or vice versa. After 24 h of virus particle incubation, the medium was removed by washing with PBS, fresh medium was added and cells were post-transduced with/without the addition of *T. officinale* extract. Luminescence was detected within 1 h using the one-step luciferase reagent from BPS following the manufacturer´s protocol in a multiplate reader from Tecan (Tecan Group Ltd., Crailsheim, Germany); solvent control: 10% distilled water (a.d.).

### 4.8. Quantification of Cytokine Release by Multiplex Bead Technique

After 24 h SARS-CoV-2 spike pseudotyped lentivirus transduction and 60 h post-infection of A549-hACE2-TMPRSS2 cells, supernatants were collected and stored at −80 °C until analysis for cytokine secretion using the human MACSplex cytokine 12-kit (Miltenyi Biotec GmbH, Bergisch Gladbach, Germany) according to manufacturer’s protocol. Cytokines below the detection limit of 3.2 pg/mL were not included in the analysis.

### 4.9. Molecular Weight Fractionation from Plant Extracts

Extracts from dried plant leaves were prepared by adding bidistilled water (5 mL) to plant material (500 mg each). The samples were incubated in the dark at room temperature (RT) for 60 min, followed by centrifugation at 16,000g for 3 min. The supernatants were collected and membrane-filtrated (0.45 µm), resulting in the extracts. Aliquots were freeze dried for 48 h to determine their yield by weight. The extracts were then further separated in a high molecular weight (HMW) and low molecular weight (LMW) fraction, using a centrifugation tube with an insert containing a molecular weight cut-off filter (5 kDa, Sartorius Stedim Biotech, Goettingen, Germany). Each HMW fraction was purified by flushing with 20 mL of water, yielding the HMW fractions, as well as LMW. The fractions were freeze dried, their yield determined by weight and stored at −20 °C until use.

### 4.10. Determination of Cell Viability Using Trypan Blue Staining

Cell viability was assessed using the trypan blue dye exclusion test as described before [41]. Briefly, A549-hACE2-TMPRSS2 cells were cultured for 24 h, and then exposed to extracts or the solvent control (a.d.) for 84 h.

### 4.11. Statistical Analysis

Results were analyzed using the GraphPad Prism 6.0 software (La Jolla, CA, USA). Data are presented as means + SD. Statistical significance was determined by the one-way ANOVA test followed by Bonferroni correction. *P*-values < 0.05 (*) were considered statistically significant and < 0.01 (**) were considered highly statistically significant.

## 5. Conclusions

Developed vaccine candidates all aim to generate antibody (and T cell) responses against the spike protein and spike sequences from the early Wuhan strain served here as a basis [42]. However, SARS-CoV-2 is steadily mutating during continuous transmission among humans. Virus antigenic drift is clearly shown by the recent appearance of new variants. It is evolving in such a way that it may eventually be able to evade our existing therapeutic and prophylactic approaches aimed at the viral spike. A growing number of studies already report reduced efficacy of neutralizing antibodies against the SARS-CoV-2 Delta variant compared with its original form [43,44]. Interestingly, *T. officinale* showed only slightly less effectiveness against the Delta variant pseudotyped virus in vitro, and the plant extract also demonstrated effective binding inhibition of four relevant spike mutations to the human ACE2 receptor. This could be a major advantage in prevention of SARS-CoV-2 infection. Thus, the results encourage more in-depth analysis of *T. officinales*´ effectiveness and now require further confirmatory clinical evidence.

## Data Availability

Data are contained in the article and Supplementary Materials.

## Supplementary Materials

The following are available online at https://www.mdpi.com/article/10.3390/ph14101055/s1, Table S1: Chemical analysis of *T. officinale* leaf extract using UPLC-TOF-MS negative ion mode (ESI−), Table S2: Chemical analysis of *T. officinale* leaf extract using UPLC-TOF-MS positive ion mode (ESI+). Table S3: Chemical analysis of *C. intybus* leaf extract using UPLC-TOF-MS negative ion mode (ESI−), Table S4: Chemical analysis of *C. intybus* leaf extract using UPLC-TOF-MS positive ion mode (ESI+).
