# Supplementary material for: In Vitro Effect of Taraxacum officinale Leaf Aqueous Extract on the Interaction between ACE2 Cell Surface Receptor and SARS-CoV-2 Spike Protein D614 and Four Mutants
Source: Pharmaceuticals (Basel). 2021 Oct 17;14(10):1055. doi: 10.3390/ph14101055 (PMC8538008; doi:10.3390/ph14101055)
Supplement: Supplementary file 1 [file pharmaceuticals-14-01055-s001.zip › Tabel_3.pdf]

Chemical analysis of *C. intybus* leaf extract using UPLC-TOF-MS negative mode (ESI-)

Compound	Adducts	Formula	m/z	Mass Error (ppm)
benzoic acid	M-H	C7H6O3	137.0249	3.6490
gallic acid	M-H	C7H6O5	137.0250	4.3788
glutamic acid	M-H	C5H9NO4	146.0454	-3.4236
tartaric acid	M-H	C4H6O6	149.0089	-2.0133
p-coumaric acid	M-H	C9H8O3	163.0397	-1.9721
caffeic acid	M-H	C9H8O4	179.0344	-3.3513
Quinic acid	M-H	C7H12O6	191.0563	1.0468
Sinapinic acid	M-H	C11H12O5	205.0605	-3.1382
Apigenin	M-H	C15H10O5	270.0519	-3.3327
4-Hydroxybenzoyl glucose	M-H	C13H16O8	299.0765	-2.3405
4-Hydroxybenzoic acid 4-O-glucoside	M-H	C13H16O8	299.0765	-2.3405
Quercitin	M-H	C15H10O7	301.0349	-1.6609
Caftaric acid	M-H	C13H12O9	304.0411	0.6430
Chlorogenic acid isomers	M-H	C16H18O9	353.0882	1.1329
Syringin	M-H, 2M-H	C17H24O9	371.1342	-0.9281
Kaempferol-7-O-glucoside	M-H, 2M-H	C21H20O11	447.0926	-1.5657
Kaempferol-3O-glucoside	M-H	C21H20O12	447.0926	-1.5657
Luteolin-7-O-glucopyranoside	M-H	C21H20O13	447.0926	-1.5657
Chicoric acid	M-H, 2M-H	C22H18O12	473.0729	0.8455
Quercitin-3-glucuronide	M-H	C21H18O13	477.0684	1.8865
Dicaffeoyl quinic acid isomers	M-H	C25H24O12	515.1203	1.5530
Rutin	M-H	C27H30O16	609.1439	-3.6116
