# Supplementary material for: In Vitro Effect of Taraxacum officinale Leaf Aqueous Extract on the Interaction between ACE2 Cell Surface Receptor and SARS-CoV-2 Spike Protein D614 and Four Mutants
Source: Pharmaceuticals (Basel). 2021 Oct 17;14(10):1055. doi: 10.3390/ph14101055 (PMC8538008; doi:10.3390/ph14101055)
Supplement: Supplementary file 1 [file pharmaceuticals-14-01055-s001.zip › Table_1.pdf]

Chemical analysis of *T. officinale* leaf extract using UPLC-TOF-MS negative ion mode (ESI-)

Compound	Adducts	Formula	m/z	Mass Error (ppm)
4-Methoxyphenoxyacetic acid	M-H2O-H	C9H10O4	163.0396	-2.6145
p-coumaric acid	M-H	C9H8O3	163.0396	-2.9362
5,8-dihydroxycoumarin	M-H	C9H6O4	177.0187	-3.6158
6,7-Dihydroxycoumarin	M-H	C9H6O4	177.0187	-3.6158
5,7-Dihydroxycoumarin	M-H	C9H6O4	177.0187	-3.6158
Citric acid	M-H2O-H, M-H	C6H8O7	191.0194	-1.9561
4-Methylesculetin	M-H	C10H8O4	191.0344	-2.7850
5,7-Dihydroxy-4-methylcoumarin	M-H	C10H8O4	191.0344	-2.7850
Quinic acid	M-H	C7H12O6	191.0560	-0.5738
Sinapinic acid	M-H2O-H	C11H12O5	205.0503	-1.3059
Pro-Glu	M-H2O-H	C10H16N2O5	225.0876	-2.0003
Glu-pro	M-H2O-H	C10H16N2O5	225.0876	-2.0003
Taraxinic acid	M-H	C15H18O4	261.1133	0.4091
Pratol	M-H	C16H12O4	267.0662	-0.3047
6-Hydroxy-2'-methoxyflavone	M-H	C16H12O4	267.0662	-0.3047
2-O-(alpha-D-glucopyranosyl)-D-glyceric acid	M-H	C9H16O9	267.0721	-0.2615
2-(alpha-D-mannosyl)-D-glyceric acid	M-H	C9H16O9	267.0721	-0.2615
Luteolin	M-H	C15H10O6	285.0395	-3.3762
Chrysoeriol	M-H	C16H12O6	299.0548	-4.4455
Sorbifolin	M-H	C16H12O6	299.0548	-4.4455
Hispidulin	M-H	C16H12O6	299.0548	-4.4455
Rhamnocitrin	M-H	C16H12O6	299.0548	-4.4455
4-Hydroxybenzoyl glucose	M-H	C13H16O8	299.0761	-3.8020
4-Hydroxybenzoic acid 4-O-glucoside	M-H	C13H16O8	299.0761	-3.8020
glucosyl salicylate	M-H	C13H16O8	299.0761	-3.8020
Caftaric acid	M-H	C13H12O9	311.0409	0.2301
gentisic acid 5-beta-glucoside	M-H	C13H16O9	315.0713	-2.6976
Fertaric acid	M-H2O-H, M-H	C14H14O9	325.0559	-2.3264
sinapoyltartronic acid	M-H2O-H, M-H	C14H14O9	325.0559	-2.3264
6-O-Galloylglucose	M-H	C13H16O10	331.0656	-4.4879
Glucogallin	M-H	C13H16O10	331.0656	-4.4879
Ascorbyl glucoside	M-H	C12H18O11	337.0779	0.8319
dalpatein	M-H	C18H14O7	341.0668	0.3146
Rosmarinic acid	M-H2O-H	C18H16O8	341.0668	0.3147
Chlorogenic acid isomers	M-H	C16H18O9	353.0872	-1.7756
Syringin	M-H	C17H24O9	371.1346	-0.4712
abscisic acid glucoside	M-H	C21H30O9	425.1810	-1.7007
6-Hydroxyluteolin 7-glucoside	M-H2O-H	C21H20O12	445.0776	-0.1085
Hypolaetin-8-glucoside	M-H2O-H	C21H20O12	445.0776	-0.1085
Gossypetin 8-rhamnoside	M-H2O-H	C21H20O12	445.0776	-0.1085
Genistein-7-O-glucuronide	M-H	C21H18O11	445.0779	0.6321
7-Hydroxy-3-(4-hydroxyphenyl)-4-oxo-4H-chromen-5-yl beta-D-glucopyranosiduronic acid	M-H	C21H18O11	445.0779	0.6321
apigenin 7-glucuronide	M-H	C21H18O11	445.0779	0.6321
apigenin 7-glucuronide	M-H	C21H18O11	445.0788	2.6623
Genistein-7-O-glucuronide	M-H	C21H18O11	445.0788	2.6623
Luteolin-5-O-glucopyranoside	M-H	C21H20O11	447.0935	0.5352
Kaempferol-7-O-glucoside	M-H	C21H20O11	447.0935	0.5352
Luteolin-7-O-glucopyranoside	M-H	C21H20O11	447.0935	0.5352
Carthamone	M-H, 2M-H	C21H20O11	447.0947	3.1519
Plantaginin	M-H, 2M-H	C21H20O11	447.0947	3.1519
Kaempferol-7-O-glucoside	M-H, 2M-H	C21H20O11	447.0947	3.1519
Isorhamnetin 4'-O-glucuronide	M-H2O-H	C22H20O13	473.0736	2.0915
Chicoric acid	M-H	C22H18O12	473.0738	2.6795
Carmine	M-H	C22H20O13	491.0836	0.9801
Isorhamnetin 4'-O-glucuronide	M-H	C22H20O13	491.0836	0.9801
Raffinose	M-H	C18H32O16	503.1626	1.5941
Maltotriose	M-H	C18H32O16	503.1626	1.5941
D-Cellotriose	M-H	C18H32O16	503.1626	1.5941
Isomaltotriose	M-H	C18H32O16	503.1626	1.5941
Dicaffeoylquinic acid isomers	M-H	C25H24O12	515.1199	0.7406
5,7-Dihydroxy-2-(4-hydroxyphenyl)-4-oxo-4H-chromen-3-yl 5-O-beta-D-glucopyranosyl-beta-D-xylofuranoside	M-H	C26H28O15	579.1370	2.5476
5,7-Dihydroxy-2-(4-hydroxyphenyl)-4-oxo-4H-chromen-3-yl 6-O-beta-D-xylofuranosyl-beta-D-glucopyranoside	M-H	C26H28O15	579.1370	2.5476
Luteolin 7-O-rutinoside	M-H	C27H30O15	593.1518	0.9918
Luteolin-7-O-neohesperidoside	M-H	C27H30O15	593.1518	0.9918
Kaempferol 3-O-neohesperidoside	M-H	C27H30O15	593.1518	0.9918
Multinoside A	M-H	C27H30O16	609.1470	1.4808
luteolin-7-*O*-gentiobioside	M-H	C27H30O16	609.1470	1.4808
Quercetin 3-O-rhamnoside 7-O-glucoside	M-H	C27H30O16	609.1470	1.4808
Rutin	M-H	C27H30O16	609.1470	1.4808
