# Supplementary material for: In Vitro Effect of Taraxacum officinale Leaf Aqueous Extract on the Interaction between ACE2 Cell Surface Receptor and SARS-CoV-2 Spike Protein D614 and Four Mutants
Source: Pharmaceuticals (Basel). 2021 Oct 17;14(10):1055. doi: 10.3390/ph14101055 (PMC8538008; doi:10.3390/ph14101055)
Supplement: Supplementary file 1 [file pharmaceuticals-14-01055-s001.zip › Table_2.pdf]

Chemical analysis of *T. officinale* leaf extract using UPLC-TOF-MS positive ion mode (ESI+)

Description	Adducts	Formula	m/z	Mass Error (ppm)
Malonic acid	M+H	C3H4O4	105.0185	2.3786
Hydroxypyruvic acid	M+H	C3H4O4	105.0185	2.3786
Pyroglutamic acid	M+H	C5H7NO3	130.0501	1.4459
Adenine	M+H	C5H5N5	136.0621	2.8388
Guanine	M+H	C5H5N5O	152.0571	2.6484
Hydroxycoumarin	M+H	C9H6O3	163.0391	0.8262
Umbelliferone	M+H	C9H6O3	163.0391	0.8262
4-Hydroxy-1-tetralone	M+H	C10H10O2	163.0757	2.0149
2-Hydroxy-3,4-dihydro-1(2H)-naphthalenone	M+H	C10H10O2	163.0757	2.0149
Phenylalanine	M+H	C9H11NO2	166.0863	0.0658
5-Phenyl-1H-pyrrole-2-carboxylic acid	M+H	C11H9NO2	188.0710	2.0678
6-Methyl-2-quinolinecarboxylic acid	M+H	C11H9NO2	188.0710	2.0678
Indoleacrylic acid	M+H	C11H9NO2	188.0710	2.0678
Tryptophan	M+H	C11H12N2O2	205.0972	0.0849
Favan-3-ol	M+H	C15H14O2	227.1066	-0.2026
Flavan-4-ol	M+H	C15H14O2	227.1066	-0.2026
7-Hydroxyflavan	M+H	C15H14O2	227.1066	-0.1922
Leu-pro	M+H	C11H20N2O3	229.1548	0.5797
Pro-Leu	M+H	C11H20N2O3	229.1548	0.5797
Isozaluzanin C	M+H	C15H18O3	247.1327	-0.7731
Zaluzanin C	M+H	C15H18O3	247.1327	-0.7731
Xerantholide	M+H	C15H18O3	247.1327	-0.7731
Mycosporine	M+H	C11H19NO6	262.1291	2.0839
(3S)-3-[(2-Carboxypropanoyl)oxy]-4-(trimethylammonio)butanoate	M+H	C11H19NO6	262.1291	2.0839
Adenosine	M+H	C10H13N5O4	268.1044	1.3913
vestitol	M+H	C16H16O4	273.1115	-2.4077
Guanosine	M+H	C10H13N5O5	284.0991	0.3939
Luteolin	M+H	C15H10O6	287.0553	0.9135
Kaempferol	M+H	C15H10O6	287.0553	0.9135
Aureusidin	M+H	C15H10O6	287.0553	0.9135
Maritimetin	M+H	C15H10O6	287.0553	0.9135
Datiscetin	M+H	C15H10O6	287.0553	0.9135
Isoscutellarein	M+H	C15H10O6	287.0553	0.9135
Scutellarein	M+H	C15H10O6	287.0553	0.9135
Kaempferol	M+H	C15H10O6	287.0555	1.6963
Datiscetin	M+H	C15H10O6	287.0555	1.6963
Luteolin	M+H	C15H10O6	287.0555	1.6963
Pro-arg	M+Na	C11H21N5O3	294.1548	4.0663
Arg-pro	M+Na	C11H21N5O3	294.1548	4.0663
Caftaric acid	M+Na	C13H12O9	335.0374	0.0779
1-O-(4-coumaroyl)-beta-D-glucose	M+Na	C15H18O8	349.0889	-1.5590
Melilotoside	M+Na	C15H18O8	349.0889	-1.5590
p-Coumaric acid glucoside	M+Na	C15H18O8	349.0889	-1.5590
Chlorogenic acid isomers	M+H	C16H18O9	355.1019	-1.3468
Lactulose	M+Na	C12H22O11	365.1070	4.5663
Melibiose	M+Na	C12H22O11	365.1070	4.5663
Trehalulose	M+Na	C12H22O11	365.1070	4.5663
Cellobiose	M+Na	C12H22O11	365.1070	4.5663
Gentiobiose	M+Na	C12H22O11	365.1070	4.5663
Trehalose	M+Na	C12H22O11	365.1070	4.5663
Xanthogalenol	M+Na	C21H22O5	377.1343	-4.5530
Xanthohumol	M+Na	C21H22O5	377.1343	-4.5530
Syringin	M+Na	C17H24O9	395.1315	0.6421
7-deoxyloganin	M+Na, M+K	C17H26O9	397.1473	1.1550
8-Epideoxyloganin	M+Na, M+K	C17H26O9	397.1473	1.1550
Rehmaionoside C	M+Na	C19H32O8	411.1991	0.5149
grandidentatin	M+Na, M+K	C21H28O9	447.1628	0.4946
Plantaginin	M+H	C21H20O11	449.1080	0.2971
Isoorientin	M+H	C21H20O11	449.1080	0.2971
galuteolin	M+H	C21H20O11	449.1080	0.2971
Trifolin	M+H	C21H20O11	449.1087	1.9134
Kaempferol-3-glucoside	M+H	C21H20O11	449.1087	1.9134
abscisic acid glucusiode	M+Na, M+K	C21H30O9	449.1785	0.6502
Zizybeoside I	M+K	C19H28O11	471.1267	0.9650
Sakuranin	M+Na	C22H24O10	471.1267	1.2707
Chicoric acid	M+Na	C22H18O12	497.0697	1.3566
Kaempferol 3-O-neohesperidoside	M+H	C27H30O15	595.1665	1.3045
5,7-Dihydroxy-2-(4-hydroxyphenyl)-4-oxo-4H-chromen-3-yl 6-O-(6-deoxyhe)	M+H	C27H30O15	595.1665	1.3045
3,5-Dihydroxy-2-(4-hydroxyphenyl)-4-oxo-4H-chromen-7-yl 2-O-(6-deoxyhe)	M+H	C27H30O15	595.1665	1.3045
Isorhamnetin 3-arabino-glucoside	M+H	C27H30O16	611.1613	0.9870
Multinoside A	M+H	C27H30O16	611.1613	0.9870
Rutin	M+H	C27H30O16	611.1613	0.9870
