# Supplementary material for: In Vitro Effect of Taraxacum officinale Leaf Aqueous Extract on the Interaction between ACE2 Cell Surface Receptor and SARS-CoV-2 Spike Protein D614 and Four Mutants
Source: Pharmaceuticals (Basel). 2021 Oct 17;14(10):1055. doi: 10.3390/ph14101055 (PMC8538008; doi:10.3390/ph14101055)
Supplement: Supplementary file 1 [file pharmaceuticals-14-01055-s001.zip › Table_4.pdf]

Chemical analysis of *C. intybus* leaf extract using UPLC-TOF-MS positive mode (ESI+)

Description	Adducts	Formula	m/z	Mass Error (ppm)
Alanine	M+H	C3H7NO2	90.0554	4.4417
Asparagine	M+H	C4H8N2O3	133.0610	1.5030
Glutamine	M+H	C5H10N2O3	147.0771	4.7594
Tartaric acid	M+H	C4H6O6	151.0231	-3.9729
caffeic acid	M+H, M+Na	C9H8O4	181.0489	-3.3140
Fructose	M+H	C6H12O6	181.0701	-3.3136
Glucose	M+H	C6H12O6	181.0714	3.8659
Tyrosine	M+H	C9H11NO3	182.0754	-0.5492
Favan-3-ol	M+H	C15H14O2	227.1070	1.3210
Flavan-4-ol	M+H	C15H14O2	227.1070	1.3210
7-Hydroxyflavan	M+H	C15H14O2	227.1070	1.3210
Caftaric acid	M+H	C13H12O9	313.0558	1.2778
Sucrose	M+H	C12H22O11	343.1219	-4.6630
p-Coumaric acid glucoside	M+H, M+Na	C15H18O8	327.1069	-1.5286
Chlorogenic acid isomers	M+H	C16H18O9	355.1017	-0.7836
Cyanidine-3-*O*-glucoside	M+	C21H21O11	449.1078	-0.6679
Kaempferol-3-*O*-glucoside	M+H	C21H20O11	449.1085	1.4681
Quercitin-3-glucuronide	M+H	C21H18O13	478.0733	-2.9284
Chicoric acid	M+H, M+Na	C22H18O12	497.0693	0.5519
Dicaffeoyl quinic acid isomers	M+H	C25H24O12	517.1339	-0.3867
Rutin	M+H	C27H30O16	611.1634	-2.4491
